# Production of Clinical-Grade SARS-CoV-2 Spike Ferritin Nanoparticle Protein Immunogen by Transient Transfection

**DOI:** 10.3390/vaccines13101041

**Published:** 2025-10-09

**Authors:** Agnes Hajduczki, William C. Chang, Rafael De La Barrera, James F. Wood, Wei-Hung Chen, Elizabeth J. Martinez, Jaime L. Jensen, Rajeshwer S. Sankhala, Clayton Smith, Alexander Anderson, Elaine B. Morrison, Caroline E. Peterson, Phyllis A. Rees, Sandrine Soman, Caitlin Kuklis, Aslaa Ahmed, Jocelyn King, Farooq Nasar, Courtney Corbitt, Misook Choe, Paul V. Thomas, Michelle Zemil, Lindsay Wieczorek, Victoria R. Polonis, Helen M. Dooley, John R. Mascola, Natalie de Val, Gary R. Matyas, Mangala Rao, Gregory D. Gromowski, Kayvon Modjarrad, Sandhya Vasan, Jeffrey W. Froude, Nelson L. Michael, M. Gordon Joyce, Stasya Zarling

**Affiliations:** 1 Viral Diseases Program, Walter Reed Army Institute of Research, Silver Spring, MD 20910, USA; ahajduczki@global-id.org (A.H.); willcchang@gmail.com (W.C.C.); jjensen@global-id.org (J.L.J.);; 2 Henry M. Jackson Foundation for the Advancement of Military Medicine, Bethesda, MD 20817, USAsvasan@global-id.org (S.V.); 3 Pilot Bioproduction Facility, Walter Reed Army Institute of Research, Silver Spring, MD 20910, USA; 4 Center for Molecular Microscopy, Center for Cancer Research, National Cancer Institute, National Institutes of Health, Frederick, MD 21702, USA; 5 Cancer Research Technology Program, Frederick National Laboratory for Cancer Research, Frederick, MD 21702, USA; 6 U.S. Military HIV Research Program, Walter Reed Army Institute of Research, Silver Spring, MD 20910, USAgmatyas@hivresearch.org (G.R.M.);; 7 Institute of Marine and Environmental Technology, University of Maryland School of Medicine, Baltimore, MD 21202, USA; hdooley@som.umaryland.edu; 8 Vaccine Research Center, National Institute of Allergy and Infectious Diseases, NIH, Bethesda, MD 20852, USA; 9 Center for Infectious Diseases Research, Walter Reed Army Institute of Research, Silver Spring, MD 20910, USA

**Keywords:** CGMP production, adjuvanted vaccine, recombinant protein vaccine, SARS-CoV-2, COVID-19, ALFQ adjuvant, transient transfection, structure-based vaccine design, pandemic preparedness

## Abstract

**Background/Objectives**: In response to the COVID-19 pandemic, we developed a vaccine candidate against SARS-CoV-2. Spike Ferritin Nanoparticle (SpFN) comprises 24 identical prefusion-stabilized spike proteins anchored to a self-assembled nanoparticle. Organized along the three-fold axis of the ferritin particle, eight SARS-CoV-2 spike trimers are presented per nanoparticle. **Methods**: Here, we describe the CGMP processes for manufacturing SpFN using transient transfection of Expi293F cells. **Results**: The final yield of SpFN was ~10 mg per liter of media supernatant. The resulting protein is stable in cold storage for two years at −20 °C, as well as for a month at room temperature, and can withstand multiple freeze/thaw cycles. SpFN material produced using the CGMP protocols adjuvanted with Army Liposomal Formulation-QS-21 (ALFQ) elicited potent neutralizing antibodies against WA-1, Alpha, Beta, and Delta variants in mice as measured by a pseudovirus neutralization assay. **Conclusions**: This work demonstrates rapid development and scaled-up production of clinical-grade SARS-CoV-2 vaccine protein material, allowing permissive storage and transport conditions, and serves as a framework for recombinant protein production for future emergent pathogens.

## 1. Introduction

Coronavirus disease 2019 (COVID-19) precipitated a pandemic in 2020, prompting a global effort to rapidly develop vaccines and therapeutics at record speed while maintaining all regulatory requirements for safety and efficacy milestones. mRNA-based vaccines, which can be quickly manufactured at scale, were the first to be available, and a set of mRNA-based vaccines were the first to secure emergency use authorization by the US FDA [1,2,3,4,5,6,7]. In contrast, protein-based vaccines require additional steps to demonstrate batch-to-batch consistency in antigenicity, purity, and stability. At this time there are over ten recombinant-based protein vaccines approved and in human use, beginning with the hepatitis B surface antigen in 1984 [8,9] to the most recent respiratory syncytial virus fusion glycoprotein in 2024 [10]. Protein-based vaccines are typically adjuvanted, and the antigen–adjuvant combination can elicit an immune response similar to or better than nucleic acid-based vaccines [11,12,13,14] while having desirable parameters such as elicitation of higher antibody titers after the primary immunization, dose-sparing, reduced reactogenicity [15], and simpler storage requirements [16], making them a desirable addition to the public-health vaccine arsenal. The long-standing history of protein-based vaccine use in humans also presents a reassuring option to communities that are reticent to the adoption of mRNA vaccine technologies, with the Novavax protein-based COVID-19 vaccine as one successful example [17,18,19].

Protein-based vaccines are intended to mimic the native conformation of the target protein immunogen in a soluble form. In the case of enveloped viruses, the preferred vaccine antigen is typically one of the pathogen’s surface glycoproteins. In the absence of a stabilizing membrane component, it can be challenging to produce stable immunogenic and soluble molecules. Most recently, structure-based vaccine design has enabled the rational design and antigenic assessment of candidate immunogens [20,21,22,23,24]. Similar to other type I fusion glycoproteins, the SARS-CoV-2 spike is a trimer with complex interfaces between protomers and undergoes significant structural rearrangements during the viral fusion process. In many human pathogen examples, viral type I fusion glycoproteins in the prefusion form present neutralizing epitopes that are hidden in the postfusion form. Spike can be engineered to maintain the prefusion structural form to be used as a stable and homogenous vaccine candidate, which can enhance the elicitation of a protective immune response.

**Figure 1:**
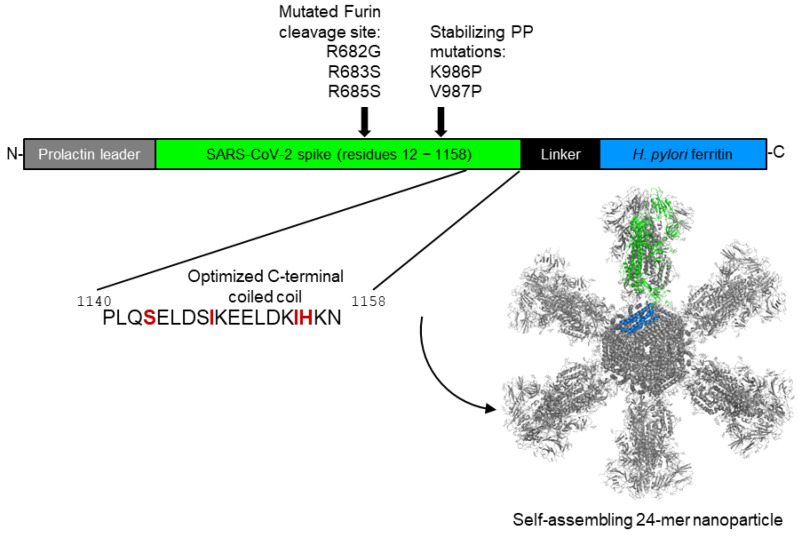
SpFN construct design: Genomic organization and the resulting protein structure. SpFN includes 1147 amino acids (87%) of the SARS-CoV-2 spike (total of 1318 amino acids). Within the spike component of SpFN, the furin cleavage site is altered by incorporating R682G, R683S, and R685S mutations, and K986 and V987 are altered to proline to enable stabilization of the spike in its prefusion form (S-2P). In the nanoparticle structure model (**lower right**), a single monomer is shown in color, with the spike component colored green and the ferritin monomer colored blue. For clarity, the spike that would project out of the page is not depicted.

A newer rational immunogen design approach involves linking of antigens to self-assembling nanoparticles. Presenting the viral molecules in an ordered array can improve the stability and immune recognition of a given antigen. In numerous cases, nanoparticle presentation has resulted in improved and broader neutralizing immune responses via improved T cell responses and B cell receptor cross-linking. One lead example is the *Helicobacter pylori* ferritin nanoparticle molecule, which has been used to generate a set of influenza and Epstein–Barr virus vaccines with several candidates in clinical trials [25,26,27]. Based on this work, we developed a series of candidate vaccines genetically fusing all or parts of the SARS-CoV-2 spike protein onto this self-assembling nanoparticle scaffold platform and selected a lead candidate, Spike Ferritin Nanoparticle (SpFN) construct 1B-06-PL [28], a 24-subunit ferritin nanoparticle displaying eight prefusion-stabilized SARS-CoV-2 spike trimers (Figure 1 and Figure S1), to advance to Current Good Manufacturing Practice (CGMP) manufacturing. This decision was based on pre-clinical studies showing favorable stability and nanoparticle formation and elicitation of potent immune responses and protective efficacy in rodent and non-human primate studies [28,29,30]. SpFN also appeared to present the fewest anticipated complications in translating to a large-scale CGMP manufacturing strategy. In part to expedite development efforts during the early stages of the pandemic, the vaccine candidate was produced using transient transfection of Expi293F cells. In our pre-clinical studies and phase I clinical trial [31], we paired SpFN with the adjuvant Army Liposomal Formulation-QS-21 (ALFQ), an adjuvant containing a mixture of saturated phospholipids, cholesterol, monophosphoryl lipid A, and QS21 saponin [32].

Here, we detail the CGMP production of SpFN and characterize the resulting drug substance (DS) and drug product (DP) materials through a set of stability and immunogenicity studies as a framework for future protein–nanoparticle vaccine development efforts.

## 2. Materials and Methods

### 2.1. SpFN Construct Design, Gene Synthesis, and Cloning

Upon release of the first SARS-CoV-2 sequence on 10 January 2020, initial constructs based on portions of spike protein linked to ferritin were designed as previously described [28]. Subsequent iterative immunogen design and optimization utilized atomic models of the SARS-CoV-2 S-2P trimer structure PDB: 6VSB and PDB: 3BVE for the *Helicobacter pylori* ferritin [24]. Visual analysis and figure generation were conducted using PyMOL V2.3.2 (Schrödinger, LLC, New York City, NY, USA).

Gene synthesis and cloning were described previously [28], as the pCoV1B-06-PL design was part of a concerted immunogen design and multi-construct assessment effort. Briefly, an initial construct named pCoV1B-05 was created utilizing DNA encoding the SARS-CoV-2 Wuhan S-2P with a native leader sequence and *Helicobacter pylori* ferritin within a pCMVR plasmid. The pCoV1B-05 DNA sequence insert was adjusted to introduce restriction sites KpnI and BamHI to allow replacement of S-2P residues 1140–1158 and the ferritin linker sequence. A subsequent pCoV1B-06 construct with an optimized ferritin linker sequence was generated from pCoV1B-05 by use of gBlock (Integrated DNA Technologies, Coralville, IA, USA) PCR amplification, followed by restriction digestion and ligation. In a later step, the native leader was replaced with the prolactin leader sequence, generating the final SpFN (pCoV1B-06-PL) construct (Figure S1).

### 2.2. SpFN Plasmid Preparation for CGMP Transfection

As pre-clinical assessments of lab-grade SpFN and other immunogens were ongoing in March–June 2020, to expedite preparations for the CGMP production, a set of three lab-produced plasmids was provided to Aldevron (Fargo, ND, USA) to enable preparation of plasmid for CGMP production. These plasmids included an RBD-Ferritin construct (pCoV131), containing the receptor binding domain (RBD) of spike protein fused to ferritin, an RBD-NTD-Ferritin construct (pCoV146), with RBD and the N-terminal domain (NTD) of the spike protein fused sequentially to ferritin, and SpFN [28] that were transfected into *E. coli* (NEB^®^ Stable Competent *E. coli* (High Efficiency)), from which master cell banks were generated. From these three plasmids, SpFN was selected to move forward based on pre-clinical evaluation. A GMP-source grade plasmid for SpFN was produced and purified by Aldevron and provided to the WRAIR team. CGMP-source production uses many of the quality controls required for CGMP material, including traceability, document control, and material qualities, while providing a faster, more cost-effective option for the transfection plasmid, which is an ancillary material in the production process. To ensure CGMP-source quality, the plasmid was assessed for absorbance 260/280 ratio purity, solution appearance, DNA homogeneity, endotoxin levels, residual host genomic DNA, residual host protein, residual host RNA, sterility, osmolality, pH, conductivity, and mycoplasma contamination, and the sequence was verified by DNA sequencing; all parameters fell within Aldevron’s GMP-source plasmid quality specifications. SpFN plasmid was stored at 5 mg/mL concentration in a final buffer of Multi-Compendial grade 10 mM Tris, 1.0 mM EDTA, pH 8.0, at −80 °C prior to use.

### 2.3. SpFN CGMP Drug Substance and Drug Product Production

#### 2.3.1. Cell Growth, Transfection, and Harvest

**Figure 2:**
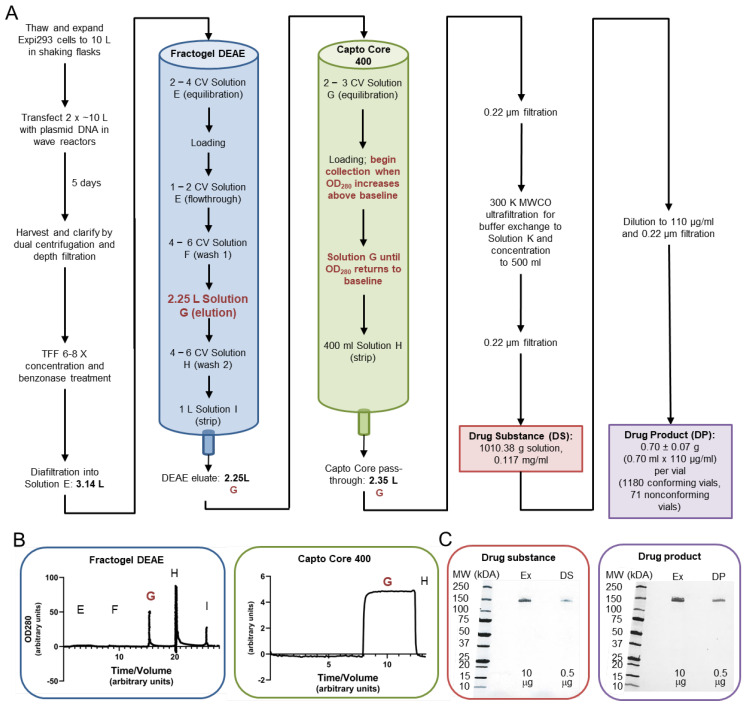
SpFN CGMP production. (**A**) Flow chart of the CGMP manufacturing process for SpFN, including transfection, growth, protein purification, diafiltration, and vialing. Red text indicates fractions from columns that proceeded to further purification. (**B**) Chromatogram traces for the CGMP Fractogel DEAE and Capto Core 400 purification steps. Respective steps as shown in panel (**A**) are indicated by the solution designation (Solution E, etc.). (**C**) SDS-PAGE for CGMP DS and DP. “Ex” designates a product from the development-grade production run; DS = drug substance and DP = drug product.

In the following production, comprehensive documentation was recorded for the CGMP processes, including batch records and standard operating procedures, as well as records of deviations, investigations, and corrective actions. All materials used in the production process were CGMP-quality materials. One vial (1.1 mL) of Expi293F HEK (CGMP banked, ThermoFisher, Waltham, MA, USA) was thawed and transferred to a 125 mL shake flask, to which 24 mL of pre-warmed Expi293 Expression media (Gibco, Waltham, MA, USA) was added. The flask was incubated at 37 ± 1 °C, 8 ± 1% CO_2_ with orbital shaking at 100 rpm for 4 d. These cells were expanded in shake flasks to 10 L over 4 additional passages of 3–4 days each. During each passage, cells were grown to reach a target cell density of 3–5 × 10^6^ cells/mL from a starting cell density of 0.3–0.4 × 10^6^ cells/mL at each passage. Cell density, viability, and media chemistry parameters (cell diameter, pH, osmolarity, and levels of glucose, lactate, glutamine, glutamate, ammonia, _P_O_2_, and _P_CO_2_) were analyzed on days 2–3 per passage and each day during expression (Nova Bioprofile Flex 2, Nova Biomedical, Waltham, MA, USA). At passage 5, cells were counted, and 2.0–2.5 × 10^10^ total cells per bag were aseptically transferred into 2 × 22 L Wave bioreactor bags (Cytiva, Marlborough, MA, USA), each containing 1 L of pre-warmed (37 ± 1 °C) Expi293 expression media (Gibco). Additional pre-warmed media was added via aseptic transfer following cell addition to reach 9 kg total weight in each bag measured by the Wave reactor loadcell, resulting in a final target cell density of 2.0–2.5 × 10^6^ cells/mL. The transfection mixture was prepared as follows: 4 mL of a 5 mg/mL solution of pCoV1B-06-PL DNA (Aldevron) was added to 996 mL phosphate-buffered saline (PBS); in a separate bottle 60 mL of Turbo293 transfection reagent (SPEED Biosystems, Gaithersburg, MD, USA) was added to 940 mL PBS. The 1 L pCoV1B-06-PL DNA-PBS mix and 1 L transfection reagent-PBS mix were combined and incubated at room temperature, in low light, for 15 min. The DNA-transfection reagent mixture was divided in two and aseptically transferred to the two wave bags (Cytiva) for a total of 10.0–10.1 kg/bag. The Wave Bioreactors were set to rocking at 22 rpm on the Wave Platform (WAVE 25 System (GE)) at the reduced temperature of 34 °C. After transfection, the culture was monitored daily for cell viability and density, and on days 3–5, SpFN concentration by qSEC, SDS-PAGE, Octet biolayer interferometry (BLI), and ELISA was performed. At 120 ± 4 h (~5 days), the culture was aseptically harvested and pooled into a 20 L BioProcess Container (Nalgene, Rochester, NY, USA), and samples of unclarified bulk harvest were collected to test for adventitious agents and mycoplasma in support of product release. The unclarified bulk harvest product was then centrifuged in 1 L centrifuge bottles at 2900× *g* for 30 min at 10 °C, the culture supernatant was collected, and centrifugation was repeated. Following centrifugation, culture supernatant was then passed through a 0.65 + 0.45 µm Sartobran clarification filter (Sartorius, Göttingen, Germany) into a sterile 20 L BioProcess Container. Samples were collected from the clarified harvest material for analysis by qSEC, SDS-PAGE, BLI, and ELISA (Figure 2).

#### 2.3.2. Bulk Drug Substance Production: Concentration, Diafiltration, Column Chromatography, and Filtration

The clarified harvest material (18.96 L) was concentrated by Tangential Flow Filtration (TFF) ~5.5-fold with a Repligen UF Hollow Fiber Module (Repligen, Waltham, MA, USA), using a 500 kDa MWCO in a Repligen KMPi TFF system at 800–1200 mL/min, maintaining a retentate pressure of ≤15 PSIG to a final volume of 3.4 L. Benzonase (EMD Millipore, Burlington, MA, USA) and MgCl_2_ (Spectrum Chemical, Brunswick, NJ, USA) were added to the concentrate to a final concentration of 50 units/mL and 2 mM, respectively, and mixed slowly (100–200 mL/min) using the TFF system with retentate lines closed and no backpressure for at least 120 min. Following Benzonase treatment, the concentrated material was diafiltered into Solution E (50 mM Tris, 50 mM NaCl, pH 8.0). During the diafiltration step, 6 dia-volumes (20.50 L) of Solution E were continuously added to the TFF system operating the pump at 800–1200 mL/min while maintaining a retentate volume of 3–4 L. Dialysis was completed when the pH of the filtrate measured 8.0 ± 0.1. Samples for analysis of protein concentration by OD280, purity by SDS-PAGE, endotoxin levels, and qSEC were taken throughout this process. Concentrated/dialyzed material was filtered using a Sartobran P 0.22 µm filtration unit (Sartorius, Göttingen, Germany).

Purification of SpFN was performed via 2-step chromatography, Fractogel DEAE (bind/elute) and Capto Core 400 (flow-through). Fractogel DEAE resin (Millipore Sigma Burlington, MA, USA) was packed on a 9 cm diameter × 15 cm ± 1 cm bed height column, sanitized, and then pre-equilibrated with 2–4 column volumes (CV) of Solution E prior to sample loading. The concentrated/dialyzed SpFN material was loaded on the Fractogel DEAE column at 170 mL/min; 500 mL of Solution E was added to the bottle towards the end of loading to facilitate uptake of the last 100–300 mL of concentrated/dialyzed material. The column was then washed with 1–2 CV of Solution E, followed by 4–6 CV of Solution F (50 mM Tris, 120 mM NaCl, pH 8.0) (wash 1). SpFN was eluted from the column with Solution G (50 mM Tris, 200 mM NaCl, pH 8.0) (elution); collection of the elution peak was initiated when the OD280 measurement increased over baseline, and collection was continued until absorbance returned to baseline (2.25 L elution pool volume). The column was washed with 4–6 CV of Solution H (50 mM Tris, 500 mM NaCl, pH 8.0) (wash 2), followed by a final strip with 1 L of Solution I (50 mM Tris, 1 M NaCl, pH 8.0) (strip). OD280 was monitored throughout the process, each flow-through was collected, and samples were removed for analysis by SDS-PAGE, qSEC, and, for equilibration and elution steps, LAL assay for endotoxin quantification.

For further purification, the elution from the Fractogel DEAE column was then passed through a Capto Core 400 column (Cytiva). Before applying the DEAE elution, the Capto Core 400 column (6 cm diameter × 10 ± 1 cm bed height) was sanitized and then equilibrated with Solution G, and the flow-through was assessed for endotoxin. The Fractogel DEAE SpFN elution pool was then loaded onto the Capto Core 400 column at 100 mL/min, monitoring OD280 during the run, and collecting the pass-through once the OD280 began to increase above baseline. Solution G was loaded onto the column at the same rate, continuing to collect the flowthrough until the OD280 peak had returned to baseline. The column was then stripped with 400 mL of Solution I. The Capto Core flow-through was then filtered using a Millipak 0.22 µm filtration unit (Millipore). OD280 of this filtered pool was measured to estimate protein concentration, and samples were taken for SDS-PAGE, qSEC, and LAL endotoxin assays.

A Repligen 300 K MWCO UF Module ultrafiltration system was cleaned and sanitized in preparation for final concentration and dialysis of the Capto Core 400 pool containing the SpFN protein. The elution pool was concentrated to 500 mL at a flow rate of 200 mL/min and inlet back pressure of 10 psi. Solution K (20 mM Na phosphate, 100 mM NaCl, 5% sucrose, 0.01% (*w*/*v*) poloxamer 188, Pluronic F-68 Polyol (MP Biomedicals, Irvine, CA, USA), at pH 7.2) was added to this system while maintaining a hold-up volume of 400–600 mL until 3 L of Solution K had been added. The dialyzed protein was then recovered from the UF system by reversing the pump and emptying the lines into the retentate bottle. Volume and OD280 of the final concentrated and dialyzed SpFN were measured to calculate protein concentration and total protein. The purified bulk SpFN was then filtered using a Millipak 0.22 µm filtration unit into a sterile, pyrogen-free Nalgene bottle. Samples of the filtered purified bulk protein and the final formulation buffer (Solution K) were then obtained aseptically for WRAIR QC release testing (Figure 2 and Figure S3, Table 1).

#### 2.3.3. Formulation and Vialing of SpFN Drug Product

Bulk Drug Substance concentration was assessed by qSEC to calculate dilution with Final Formulation Buffer (Solution K) to a final concentration of 110 µg/mL. The formulated material was filtered by a Millipak-20, 0.22 µm filter (Millipore) into a sterile 1 L PETG bottle (Nalgene) (875 mL). Washed and sterilized 2 mL Wheaton serum tubing vials (DWK Life Sciences, Vineland, NJ, USA) were filled to a target volume of 0.7 mL, stoppered (FluroTec-coated, 13 mm, West Pharmaceutical Services, Exton, PA, USA), and crimped (Plastic Top Flip-Off Crimp Seals, Grey, 13 mm, West). Crimped vials were submitted for QC inspection for defects and particulates with a final lot size of 1180 conforming vials, with 40 conforming vials maintained as reserve (Figure 2).

#### 2.3.4. Production of Development Grade and Reference Standard Material

Development-grade SpFN was produced under non-GMP conditions following a similar process as described above and previously [28]. Reference materials QRS001 and QRS002 were derived from non-GMP representative development runs produced at the 10 L and 20 L scale manufacturing process, respectively. QRS001 was derived utilizing HEK293 suspension cells (NIH, Vaccine Research Center), while QRS002 was derived utilizing Expi293F suspension cells (ThermoFisher). Both processes were similar in harvest, purification, and formulation to the process described above. Briefly, for QRS001, HEK293 cells were expanded to desired cell density at 10 L in shake flask culture in Freestyle293 Expression Medium (Gibco) at 37 °C, 8% CO_2_, >60% RH, and 120 rpm. HEK293 cells were transfected at 2 × 10^7^ cells/mL density with 20 mg/L SpFN pDNA (Aldevron) and 40 mg/L polyethylenimine (PEI 25 K, transfection grade, Kyfora Bio Polysciences, Inc, Warrington, PA, USA). QRS002 was generated from transfection of Expi293F cells, maintained in Expi293 medium in shake flask culture at 37 °C, 8% CO_2_, >60% RH, and 120 rpm. Expi293F cells were transfected with 1 mg/L SpFN pDNA (Aldevron) + 3 mL/L Turbo 293 reagent. For both QRS001 and QRS002, following transfection, the temperature was lowered, and cells were maintained at 34 °C, 8% CO_2_, 120 rpm, and >60% RH for expression and harvested at day 5 post-transfection. Harvest material was clarified by centrifugation followed by 0.65 µM + 0.45 µM depth filtration (Sartorius). Clarified material was treated with benzonase, purified by Fractogel DEAE and Capto Core 400 chromatography, and dialyzed as described above for the CGMP Bulk Drug Substance purification. Characterization of QRS001 and QRS002 demonstrated that both materials met target specifications, and both were suitable for use as reference standard material.

### 2.4. SpFN Drug Product Characterization

To ensure that the drug product maintained desirable antigenic properties, binding studies with a spike-specific antibody were performed by BLI, using a ForteBio Octet Red 96 instrument (Sartorius). ShAb02 (WRAIR), a SARS-CoV-2 RBD-specific monoclonal antibody, served as the detection reagent [33]. SpFN reference material was used as a standard. SARS-CoV-2 Spike S1-His Recombinant Protein, 40591-V08H (Sino Biological, Beijing, China), served as a positive control. PBS was used as a negative control. The reference standard and samples were tested at concentrations of 20, 10, 5, and 0 µg/mL; the positive control was tested at 20 µg/mL. Each well used in the 96-well assay plate had 150 µL volume. Anti-human IgG Fc capture (AHC) biosensors hydrated in PBS pH 7.4 (Gibco) were dipped into ShAb02 (40 µg/mL) for 180 s (loading), PBS for 30 s (baseline), sample for 180 s (association), and then PBS for 30 s (dissociation). The average of the 20, 10, and 5 µg/mL wells’ responses at 180 s after the start of the association step was reported as the average response. Relative potency (%) was reported as the sample average response divided by the reference standard average response multiplied by 100.

The stability of the SpFN recombinant protein and ALFQ in mixed vaccine formulations was assessed for holding time from dose preparation to clinical administration (25 μg and 50 μg SpFN mixed with 0.5 mL ALFQ adjuvant in a total 1.0 mL volume in vaccine formulations at room temperature (20–25 °C) for 0 and 6 + 1 h post-mixing). The ALFQ adjuvant used was pre-formulated and pre-vialed CGMP-quality material that has been used in multiple phase I clinical studies. SpFN DP (0.6 mL from 1 vial) was transferred into the vial containing ALFQ and mixed by gently swirling the vial for 2 ± 0.3 min. The mixture was incubated at room temperature for 0 h and 6 h prior to grid preparation. TEM grids were glow-discharged for 15 s. Samples were diluted by 1:2 to 1:8 with PBS, with 3.5 µL applied to the grids and allowed to sit for 30 s prior to removal by wicking with Whatman paper. The grid was “washed” with 3.5 µL of water, immediately removed by wicking, and repeated twice prior to the addition of 3.5 µL of 0.75% uranyl formate for 30 s. Almost all of the uranyl formate solution was wicked off, leaving a thin film on the grid, prior to drying and imaging at 36,000× magnification. Images were collected on a Talos L120C TEM electron microscope with a Ceta 4 k × 4 k camera and analyzed. The SpFN + ALFQ mixture was also assessed for any change in appearance (no particulates were observed), pH change (no change observed), or liposome particle size change (no change observed by DLS), with no changes detected.

Stability of the DS and DP stored under different temperatures and durations was assessed by BLI as described above.

### 2.5. Pre-Clinical Product Testing

#### 2.5.1. Immunogen–Adjuvant Preparation

The development grade SpFN was stored at 4 °C at 1 mg/mL and subsequently diluted with dPBS (Quality Biological, Inc., Gaithersburg, MD, USA) to provide 10 μg or a lower amount per 50 μL dose upon mixing with adjuvant. ALFQ (1.5×) liposomes, containing 600 μg/mL monophosphoryl 3-deacyl lipid A (3D-PHAD) and 300 μg/mL QS-21, were gently mixed by slow-speed vortex prior to use. The ALFQ material used in the mouse immunization study was prepared 2–24 h prior to antigen addition and stored at 4 °C. Antigen was added to the ALFQ, mixed by slow-speed vortex for 1 min, mixed on a roller for 15 min, and stored at 4 °C for 1 h prior to immunization. The final adjuvanted SpFN immunogens were formulated with ALFQ to contain 20 μg 3D-PHAD and 10 μg QS21 per 50 μL dose.

#### 2.5.2. Immunization of Mice with SpFN Adjuvanted with ALFQ

Research was conducted under an IACUC-approved animal use protocol in an American Association for Accreditation of Laboratory Animal Care (AAALAC) Internationally-accredited facility with a Public Health Services Animal Welfare Assurance and in compliance with the Animal Welfare Act and other federal statutes and regulations relating to laboratory animals. This research adhered to the principles stated in the Guide for the Care and Use of Laboratory Animals, NRC Publication, eighth edition, 2011. The research protocol was approved by the Institutional Animal Care and Use Committee of WRAIR. BALB/c and C57BL/6 mice were obtained from Jackson Laboratories (Bar Harbor, ME, USA). Mice were housed in the animal facility of WRAIR, accredited by AAALAC International and holds an Assurance with the National Institutes of Health Office of Laboratory Animal Welfare, and tended in accordance with local, state, federal, and institutional policies.

Three groups of C57BL/6 and three groups of BALB/c mice (n = 10/group) were used for the immunizations, each group receiving either 10 µg, 2 µg, or 0.08 µg of the development grade SpFN material (QRS002) adjuvanted with a consistent 1× dose of ALFQ containing 20 μg of 3D-PHAD and 10 μg of QS21 Mice were randomly assigned to experimental groups without pre-screening or selection based on size or other gross physical characteristics. The mice were immunized at weeks 0, 3, and 6 intramuscularly in alternating caudal thigh muscles. Blood samples were collected for analysis at weeks 2, 5, and 8. Serum was stored at 4 °C or −80 °C until analysis. Antibody responses were assessed by pseudovirus neutralization assay.

#### 2.5.3. SARS-CoV-2 and SARS-CoV-1 Pseudovirus Neutralization Analysis

Pseudovirus neutralization was performed as previously described [27]. Briefly, plasmids based on pcDNA3.4 encoding the spike proteins (S) from SARS-CoV-2 WA-1, Alpha (B.1.1.7), Beta (B.1.351), Delta (B.1.617.2), or SARS-CoV-1 (Urbani) were codon optimized for mammalian expression. An 18-amino acid endoplasmic reticulum retention signal was removed from the cytoplasmic tail to allow increased incorporation of S into pseudovirus particles. Virions pseudotyped with the vesicular stomatitis virus (VSV) G protein were used as a control. The pseudoviruses were produced by co-transfection of HEK293T/17 cells with the SARS-CoV-2 S plasmid and an HIV-1 NL4–3 luciferase reporter plasmid (obtained through the NIH HIV Reagent Program, Division of AIDS, NIAID, NIH: Human Immunodeficiency Virus 1 (HIV-1) NL4–3 Δenv Vpr Luciferase Reporter Vector (pNL4–3.Luc.R-E-), ARP-3418, contributed by Dr. Nathaniel Landau and Aaron Diamond). For the determination of neutralization titers, mouse serum samples were serially diluted in growth medium. After the addition of the SARS-CoV-2 pseudovirus, the plates were incubated at 37 °C for 1 h. ACE2-expressing HEK293 target cells (Integral Molecular, Philadelphia, PA, USA) were added to each well (40,000/well), and the plates were incubated for 48 h. RLUs were measured with the EnVision Multimode Plate Reader (Perkin Elmer, Waltham, MA, USA) using the Bright-Glo Luciferase Assay System (Promega Corporation, Madison, WI, USA). The assay was performed in a semi-automated manner, using a robotic liquid handling system (Biomek NX^P^, Beckman Coulter, Indianapolis, IN, USA). For data analysis, neutralization dose–response curves were fitted by nonlinear regression with a five-parameter curve using the LabKey Server^®^ [34], and the final titers are reported as the reciprocal of the dilution of serum necessary to achieve 50% neutralization (ID50, 50% inhibitory dilution). Assay equivalency for SARS-CoV-2 was established by participation in the SARS-CoV-2 Neutralizing Assay Concordance Survey (SNACS) run by the Virology Quality Assurance Program and External Quality Assurance Program Oversite Laboratory (EQAPOL) at the Duke Human Vaccine Institute, sponsored through programs supported by the National Institute of Allergy and Infectious Diseases, Division of AIDS.

## 3. Results

### 3.1. SpFN Bulk and Vialed Product Concentration and Identity Determinations

**Figure 3:**
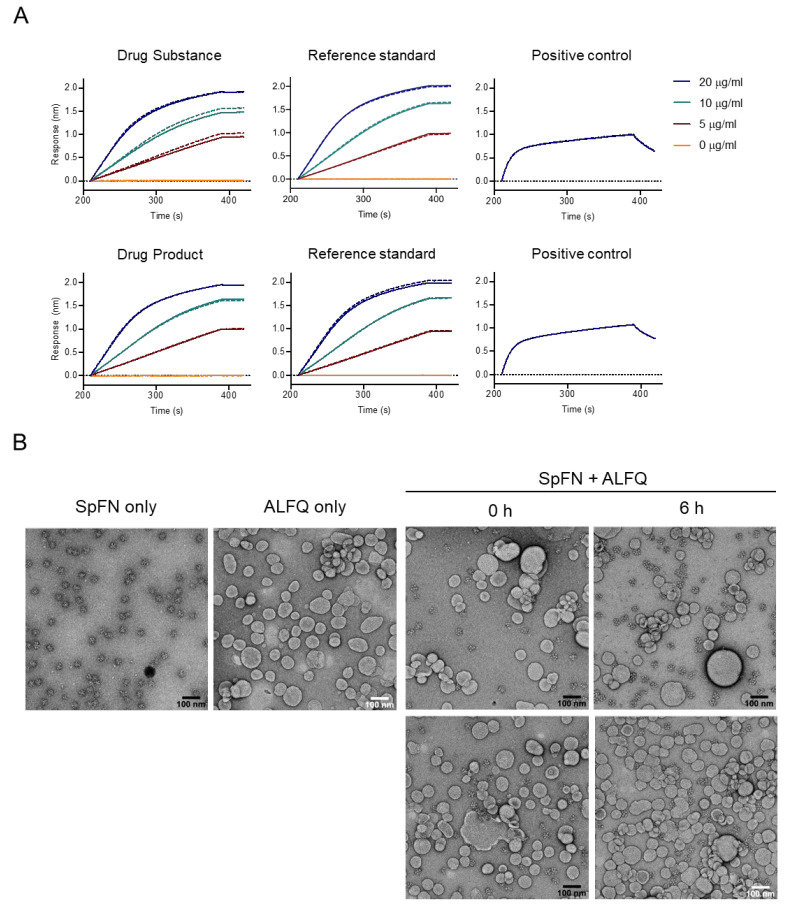
(**A**) Biolayer interferometry traces of DS (**upper row**) and DP (**lower row**) with their corresponding controls (SpFN from the development run) and positive controls (SARS-CoV-2 Spike S1-His Recombinant Protein) at day 0, binding to ShAb02. Solid and dashed lines for each color show duplicate measurements. (**B**) Negative stain electron micrographs of SpFN (25 mg/mL), ALFQ (0.5×), and a mixture of SpFN and ALFQ (12.5 mg/mL and 0.25×, respectively) at 0 h and 6 h after mixing.

In the development production run, the yield from 16.0 L cell culture was 55.47 mg (QRS002). For the CGMP preparation, the yield of SpFN DS from 18.96 L of cell culture was 118.46 mg. These are roughly similar to the 5 mg/L that was produced at laboratory scale [28]. For the initial DS and DP release criteria, identity and purity of SpFN following production were confirmed by SDS-PAGE for both the development run and the CGMP run, with a single major band observed at ~150 kDa conforming to the reference standard QRS001 (25 September 2020). Purity and quality were also assessed by qSEC, HCP, ELISA, and DLS for the CGMP run (Figure 2 and Figure S2, Table 2). Endotoxin levels of the DS were 2.6 EU/50 µg SpFN and of the DP 5.2 EU/50 µg SpFN, meeting our specification of ≤10 EU/50 µg SpFN. DS and DP were tested for binding by BLI to ShAb02 [33] alongside the reference standard and a positive control (SARS-CoV-2 Spike S1-His Recombinant Protein, 40591-V08H (Sino Biological)) to establish equivalence between the DS, DP, reference standard, and the positive control, and to establish a concentration range in which SpFN binding to ShAb02 is dose-dependent (Figure 3A). Potency was 96.1% for DS and 98.7% for DP using the ShAb02 monoclonal antibody (WRAIR) alongside the reference standard and positive control.

### 3.2. SpFN Particle Formation and SpFN/ALFQ Liposome Formation Assessment by Negative-Stain EM

SpFN DP was mixed with ALFQ, and the SpFN-ALFQ samples were diluted to 50 µg and 25 µg/mL and applied to copper grids and stained with uranyl formate stain. In all test examples, at both times, 0 h and 6 h, the SpFN nanoparticle was clearly displayed with the characteristic central ferritin ring and protruding spikes (Figure 3B) as previously described for research-grade material [28]. There was no evidence of sample decay, nanoparticle disruption, or aggregation in the samples at either of the timepoints assessed.

### 3.3. DS and DP Stability Analyses by BLI and SDS-PAGE

**Figure 4:**
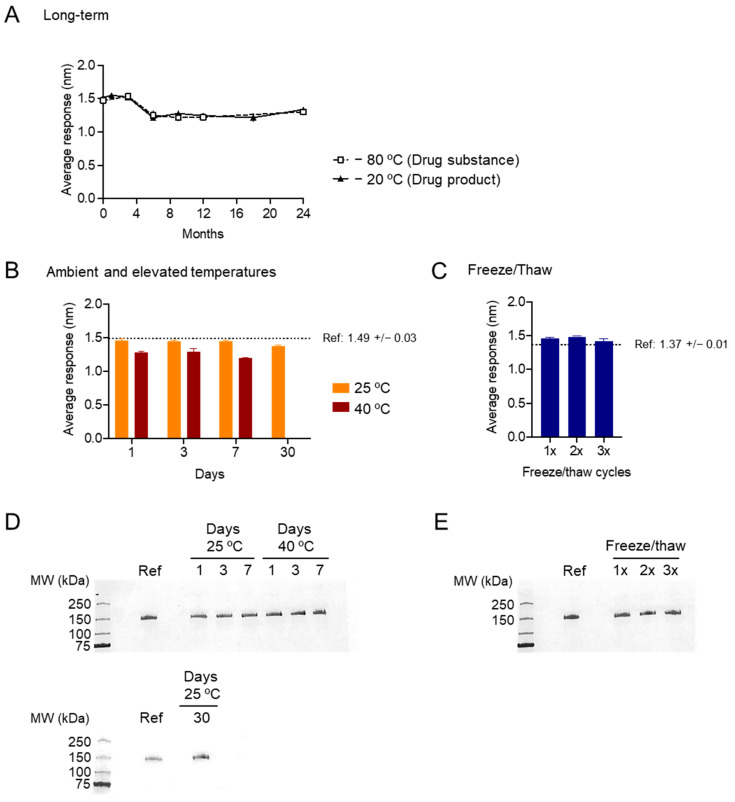
Stability of SpFN material as assayed by BLI and SDS-PAGE. (**A**) For long-term storage (up to 24 months), the drug substance was stored at −80 °C, and the drug product was stored at −20 °C. (**B**) For the ambient and elevated temperature assessments, the drug product was stored at either 25 °C for up to a month or 40 °C for up to a week. (**C**) Freeze/thaw cycles were performed on the SpFN DP. Samples were compared to a freshly thawed reference material. For all experiments shown, anti-human IgG probes (AHC) were used to capture the antibody, then the probes were immersed in 20 µg/mL, 10 µg/mL, or 5 µg/mL protein and assayed in duplicate. The average of the responses at all three concentrations at 180 s, after the start of the association step, was reported as the average response. Relative potency (%) was reported as the sample average response divided by the reference standard average response multiplied by 100. Data shown are expressed as averages with standard deviations of the values obtained at all three protein concentrations. Error bars are sometimes not visible due to small errors. (**D**) SDS-PAGE of the ambient and elevated temperature samples. Lower figure show 30 days at 25 °C. (**E**) SDS-PAGE of the freeze/thaw samples.

DS (stored at −80 °C) and DP (stored at −20 °C) were tested for potency at 0, 1, 3, 6, 9, 12, 18, and 24 months after manufacture (Figure 4A). Relative potency (against the reference standard, stored at −80 °C) was >90% over the course of the 24 months. Relative potency of DP after storage at 25 °C and 40 °C for 1, 3, 7, and (for 25 °C only) 30 days was also assessed (Figure 4B). At 25 °C, relative potency was >95% over 30 days; at 40 °C, relative potency was 85.8% after 1 day, 85.0% after 3 days, and 80.5% after 7 days. Relative potency of DP after freeze/thaw cycles was also assessed; after 1, 2, or 3 freeze/thaw cycles, relative potency was ~100% (Figure 4C). Assessment of protein size and purity by SDS-PAGE showed minimal change after storage at elevated temperatures (Figure 4D) or freeze–thaw cycles (Figure 4E).

### 3.4. In Vivo Assessment of Development-Grade SpFN

**Figure 5:**
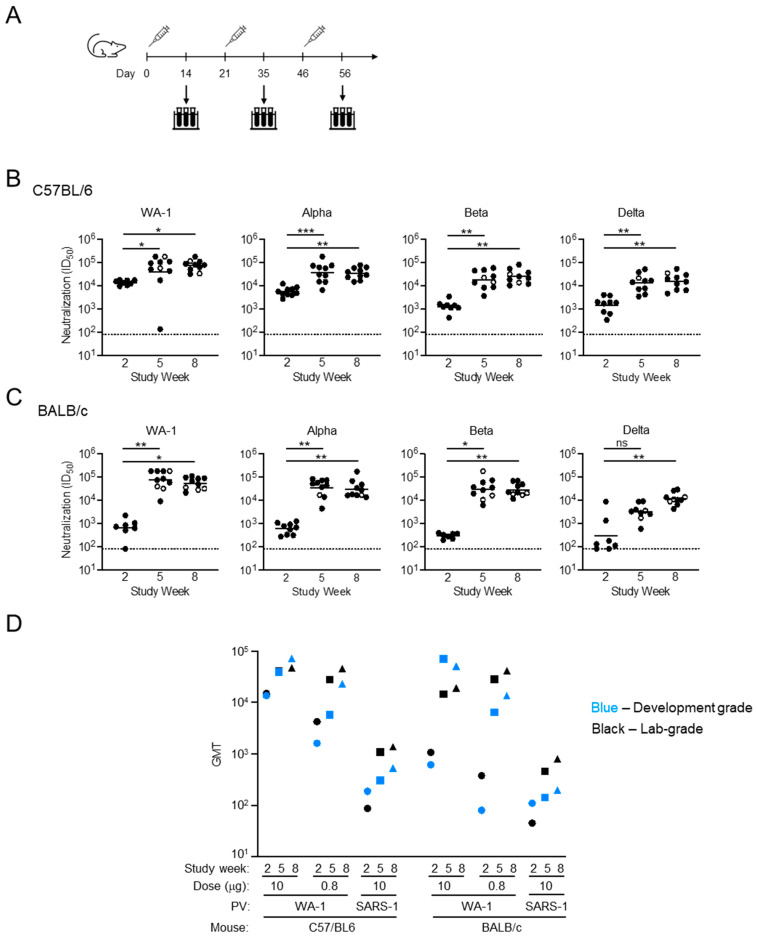
Neutralization of pseudotyped viruses by sera of mice immunized with SpFN + ALFQ. (**A**) Immunization and sample collection scheme. Arrows indicate days of blood collection. (**B**) C57BL/6 and (**C**) BALB/c mice were immunized with 10 µg of Development-Grade material, and sera samples were assayed for neutralization using SARS-CoV-2 WA-1, Alpha, Beta, and Delta pseudotyped viruses. Statistical comparisons between the timepoints (one-way ANOVA, Friedman test) were performed on the data points represented by closed circles, for which there was data for all three post-immunization timepoints. *** *p* < 0.001, ** *p* < 0.01, * *p* < 0.1, ns = not significant. Open circles are samples that were excluded from the paired statistical analysis, as they were not present at all three timepoints. (**D**) Comparison of neutralization of mice immunized by SpFN produced at development grade (blue) or lab grade (black), arranged by equivalent mouse strains and dose groups.

C57BL/6 and BALB/c mice were immunized with 10 µg, 2 µg, and 0.08 µg SpFN (QRS002 (27 October 2020)) + ALFQ Development-Grade material and monitored for neutralizing antibody production (Figure 5, Figures S2 and S4). The Development-Grade material was expressed and purified according to the CGMP protocol as the final drug product, without going forward to vialing. Mice immunized with 10 µg of SpFN + ALFQ showed a robust neutralizing response against four SARS-CoV-2 variants of concern that were assessed (Figure 5A). In the C57BL/6 mice, even after one immunization, there was a considerable response, reaching an ID50 (GMT) of >13,000 against WA-1 and between 1200 and 5400 for SARS-CoV-2 VoCs Alpha, Beta, and Delta (Figure 5B). In comparison, BALB/c mice had lower neutralization titers after the first immunization, with the highest titers against the vaccine-matched WA-1 strain at ~600 (Figure 5C). However, following the second immunization, both strains of mice reached a peak response with ID50 (GMT) of <39,000 for C57BL/6 and <70,000 for BALB/c against WA-1. Neutralizing responses against Alpha and Beta pseudoviruses resulted in an ID50 of ~35,000 in both mouse strains. For Delta, neutralization titers after the second immunization were at >13,000 and >3000 for C57BL/6 and BALB/c mice, respectively (Figure 5B,C). In the C57BL/6 mice, for all pseudoviruses except Alpha, the third immunization further improved neutralization titers, with the response against WA-1 at >71,000 being the highest among all groups. In the BALB/c groups with WA-1, Alpha, and Beta pseudoviruses, the third immunization did not increase the neutralization titers, except in the BALB/c model against Delta pseudovirus, where there was a greater than 3.5-fold increase in ID50 between the second and third collection timepoints (Figure 5B,C). For both C57BL/6 and BALB/c mice, mice immunized with development-grade SpFN stored at 4 °C over the course of this study gave geometric mean titers (GMT) against SARS-CoV-2 WA-1 and SARS-CoV-1 across timepoints and dose groups consistent with published results using research-grade SpFN [28] (Figure 5D).

## 4. Discussion

Rapid development of a CGMP vaccine product against a novel pathogen can be complicated by the limited and delayed availability of reliable pathogen-specific reagents, such as antibodies. The shark-derived antibody, MoB3-3D8-Fc (ShAb02), provided a high-affinity antigen-specific reagent at a time when SARS-CoV-2 antibodies compatible for CGMP process development were not readily available [33]. ShAb02 contains the single-chain variable domain of a new antigen receptor (VNAR) of a shark antibody, isolated from a nurse shark immunized with the SARS-CoV-2 receptor-binding domain (RBD). The VNAR domain was genetically fused to human IgG1 Fc, enabling production of a highly specific, versatile reagent that could be used in biochemical assays and was subsequently used during the characterization of the DS and DP [33]. Utilization of shark- or camelid-derived antibodies represents a means to generate necessary reagents when human antibodies are limited or absent.

Comparable to the SpFN protein produced at laboratory scale, the CGMP DS and DP showed dose-dependent binding to ShAb02 and maintained potency under different time and temperature conditions. SpFN produced in the manner described in this paper and administered to mice with ALFQ gave equivalent neutralization results as SpFN produced at research-grade (Figure 5D) [28]. Army Liposomal Formulation containing QS-21 (ALFQ), a liposomal adjuvant, outperformed the Alhydrogel adjuvant in eliciting binding and neutralizing responses in murine and non-human primate pre-clinical studies [28,35], and our group has significant experience with ALFQ [36]. Given the strong breadth, potency, and stability observed, ALFQ was selected as the adjuvant used in multiple pre-clinical studies and a human phase I clinical study. The phase I clinical trial performed using CGMP-grade SpFN and ALFQ elicited robust binding and neutralizing antibodies against SARS-CoV-2 variants, including Omicron XBB.1.5, with additional neutralization of diverse sarbecoviruses, including SARS-CoV-1 [31]. The breadth of immune response elicited by this vaccine—designed based on the WA-1 variant—against variants that emerged up to three years later highlights the SpFN platform combined with ALFQ produces an immune response with meaningful breadth of activity.

The laboratory-scale SpFN was purified using lectin affinity followed by size exclusion chromatography, similar to other viral glycoprotein vaccine candidates [13,37,38,39]. However, for scaled-up production of CGMP quality, we shifted to a purification scheme based on ion exchange chromatography and ultrafiltration. Use of ion exchange has also been successful for purification of gHgL-ferritin constructs [40]. The columns and resins used in the final protocol were selected through an iterative series of pilot expression experiments. DEAE and Capto Core columns allowed for the purification of antigenically and structurally intact immunogen. Affinity-purification resins, and, in particular, specialized lectin resins used to purify untagged proteins, are more expensive than ion-exchange resins and present novel process development risk profiles [41,42]. Size exclusion resins, while available at scale commercially, would require large CV per load volumes, presenting a challenge in fitting such a column in a facility and necessitating ultrafiltration and concentration before loading [41,42]. Innovations including single-use affinity membrane columns and engineered protein ligands may improve the cost and scalability of affinity purification, although this will vary depending on the type of vaccine being purified [43]. The protein-based SARS-CoV-2 vaccines Novavax Nuvaxovid (NVX-CoV2373) [44] and Sanofi/GSK VidPrevtyn Beta (CoV2 preS dTM B.1.351) [45,46] used affinity purification, as did all published purifications of SARS-CoV-2 S protein up until June 2021 [41]. However, for Corbevax (RBD219-N1C1), a modified RBD [47] with published adult phase 1/2 [48] and pediatric phase 2/3 [49] clinical results and pre-clinical efficacy against the Omicron BA.1 variant [50], purification methods were optimized when scaling up for CGMP production, replacing the size exclusion step used in laboratory-scale production with anion exchange chromatography specifically because of its cost and scalability [51]. Similarly, Icosavax IVX-411, an early vaccine candidate based on the same RBD-I53-50 nanoparticle design as SKYCovione (GBP510), changed cell lines and expression (transient transfection in HEK293 to stable CHO cell pools) and purification schemes (immobilized metal affinity chromatography (IMAC) purification steps replaced with hydrophobic interaction chromatography (HIC), mixed mode chromatography, and anion exchange chromatography) [52,53].

The emergence of new SARS-CoV-2 variants, such as the BA.1 Omicron lineage first detected in South Africa, emphasized the need to treat the COVID-19 pandemic as a worldwide health concern. Vaccination in low- and middle-income countries lagged behind the developed world and is still insufficient in some low-income countries. Impediments to distribution include the need to store and transport mRNA vaccines in stable, controlled conditions—many countries do not have reliable cold chain infrastructure, especially in rural areas. Protein-based vaccines and their adjuvants have less stringent storage and transport requirements, such as refrigerated (4 °C) or ambient temperatures. For example, Novavax Nuvaxovid has been assessed to be stable at 2–8 °C for 12 months (protected from light) and up to 12 h at 25 °C [16]. When tested against different potential storage and transport conditions, SpFN is comparable, retaining full potency after three freeze–thaw cycles, >95% potency after 30 days at 25 °C, and over 80% potency even after 30 days at 40 °C. ALFQ, the adjuvant paired with SpFN during clinical trials, is also stored under refrigerated (4 °C) conditions [54]. As SpFN-based constructs and ALFQ adjuvant can be readily produced to maintain efficacy against emerging variants and can be stored and transported in less stringent conditions than mRNA vaccines, they could address the continuing need for SARS-CoV-2 vaccination across the world.

Following the public availability of the SARS-CoV-2 spike protein sequence, our extensive structure-based vaccine design resulted in the SpFN molecule through the assessment and CGMP production, which took 10 months to accomplish. We have demonstrated that SpFN remains potent for several years, both as a stable immunogen, and when combined with ALFQ can elicit broadly protective antibodies against divergent VoCs. This validates the feasibility of following a similar vaccine development strategy to address future emergent pathogens.

## 5. Conclusions

We have demonstrated scaled-up production of CGMP-quality SpFN that displays stability up to two years post-production. Overall, the lab-scale and the CGMP SpFN material displayed similar and consistent structural, physical, and antigenic properties. Similar to the pre-clinical research-grade production, CGMP expression of SpFN was achieved using transient transfection of Expi293F cells. Advantages of HEK293 cell lines over other commonly used cell lines include human glycosylation patterns and post-translational modifications and reduced off-target immunogenicity [55]. Pre-clinical studies of influenza ferritin nanoparticle vaccines used expression in Freestyle HEK293 cells [56], but for SpFN, Expi293F cells consistently showed greater expression at both laboratory and manufacturing scales and thus were selected for this CGMP effort. We continued with transient transfection for CGMP expression to accelerate the availability of this vaccine candidate to address the evolving pandemic. Continuing future development using transient transfection or transitioning to a stable cell line each present their own advantages and disadvantages. Transient transfection often results in higher initial yields but requires large-scale production of CGMP-quality DNA and QC testing for residual transfection reagents. In contrast, stable cell lines would need additional and time-consuming efforts to develop but are likely to give more consistent and higher expression levels with reduced batch-to-batch variability [55,57]. Ongoing technological developments in transient transfection address some of these drawbacks, allowing the migration to stable cell development to be skipped or delayed to a later stage in development [57,58,59,60]. This becomes most relevant in the case of a rapid requirement for a product for clinical testing as occurred during the COVID-19 pandemic.

## Figures and Tables

**Table 1:** Buffers used in the purification of SpFN.

Buffer Name	Purpose	Composition
Solution A	70% Ethanol for Sanitization	70% ethanol
Solution B	6 N Hydrochloric Acid (HCl) for pH Adjustment	6 N HCl
Solution C	1 N Sodium Hydroxide (NaOH) for pH Adjustment	1 N NaOH
Solution D	Ultrafiltration System Cleaning Solution	0.5 N NaOH
Solution E	1st TFF Dialysis and Fractogel DEAE Equilibration Solution	50 mM Tris, 50 mM NaCl, pH 8.0
Solution F	Fractogel DEAE Wash Solution	50 mM Tris, 120 mM NaCl, pH 8.0
Solution G	Fractogel DEAE Elution Solution	50 mM Tris, 200 mM NaCl, pH 8.0
Solution H	Fractogel DEAE Column Solution	50 mM Tris, 500 mM NaCl, pH 8.0
Solution I	Fractogel DEAE Strip Solution	50 mM Tris, 1 M NaCl, pH 8.0
Solution J	Benzonase Treatment	200 mM MgCl_2_
Solution K	Final Formulation Buffer	20 mM sodium phosphate, 100 mM NaCl, 5% Sucrose, 0.01% (*w*/*v*) Poloxamer 188, pH 7.2
Solution L	Column Storage	20% Ethanol

**Table 2:** Characterization of SpFN DS and DP.

Test	Specification	DS Result	DP Result
Appearance, USP	Clear, colorless liquid with no turbidity and no visible particulates	Clear, colorless liquid with no turbidity and no visible particulates (PASS)	Clear, colorless liquid, with no turbidity and no visible particles (PASS)
pH, USP	6.0–8.0	pH @ 26 °C = 7.2 (PASS)	pH @ 26 °C = 7.3 (PASS)
Concentration by qSEC	>100 µg/mL	117 µg/mL (PASS)	104 µg/mL (PASS)
Purity by qSEC	≥80% total area at a retention time of 3.8 min ± 10%	92% area at a retention time of 3.8 min (PASS)	91% area at a retention time of 3.8 min (PASS)
Endotoxin Content, USP	≤10 EU/50 µg SpFN	2.6 EU/50 µg SpFN (PASS)	5.2 EU/50 µg SpFN (PASS)
Biolayer Interferometry Binding Assay	Report Result	% relative potency = 96.3%	% relative potency = 98.7%
SDS-PAGE	Comparable to Reference Standard QRS001	Comparable to ref. std. QRS001 (PASS)	Comparable to ref. std. QRS001 (PASS)
Western Blot	Comparable to reference standard QRS001, positive for ShAb02	Comparable to ref. std. QRS001, positive for ShAb02 (PASS)	Positive for ShAb02 (PASS)
Osmolarity	Report Result	399 mOsm/kg	396 mOsm/kg
Subvisible Particulate Matter by USP	Report Result	≥10 µm = 963 ≥25 µm = 223	Not tested
Product Particle Characterization by DLS	Report Result	Peak 1 = 98.8% at 35.26 d.nm Peak 2 = 1.2% at 3894 d.nm	Peak 1 = 96.8% at 35.44 d.nm Peak 2 = 3.2% at 1949 d.nm
Host Cell DNA by qPCR	Report Result	<4.27 × 10^4^ fg/mL	Not required
Residual Benzoase by ELISA	Report Result	<0.195 ng/mL (below the limit of quantification)	Not required
Residual DNA for transforming Adenoviruses	Report Result	<2.11 × 10^3^ copies/mL for both E1A and E1B	Not required
Residual pDNA by Kanamycin resistance marker	Report Result	None detected (below the limit of quantification) (PASS)	Not required
Bioburden, USP	<10 CFU/10 mL	TAMC = 0 CFU/10 mL TYMC = 0 CFU/10 mL (PASS)	Not required
Container Closure Integrity Test	No leaking containers	Not required	No leaking containers (PASS)
Sterility Qualification	No bacteriostasis or fungistasis	Not required	No bacteriostasis or fungistasis (PASS)
Sterility, USP	No growth	Not required	No growth (PASS)

## Data Availability

The original contributions presented in this study are included in this article/Supplementary Materials. Further inquiries can be directed to the corresponding authors.

## Supplementary Materials

The following supporting information can be downloaded at: https://www.mdpi.com/article/10.3390/vaccines13101041/s1. Figure S1: SpFN construct amino acid sequence. Figure S2: SpFN development run production. Figure S3: SDS-PAGE of in-process samples from the CGMP manufacturing process. Figure S4: Immunization of mice with SpFN + ALFQ.

## Abbreviations

The following abbreviations are used in this manuscript:

AIDS	acquired immunodeficiency syndrome
ALFQ	Army Liposome Formulation containing *Quillaja saponaria* saponin fraction 21
ANOVA	Analysis of Variance
CFU	Colony-Forming Units
CGMP	Current Good Manufacturing Practice
COVID-19	Coronavirus Disease 2019
CV	column volume
DEAE	diethylaminoethyl
DLS	Dynamic Light Scattering
DNA	deoxyribonucleic acid
DP	drug product
DS	drug substance
ELISA	Enzyme-Linked Immunosorbent Assay
EQAPOL	External Quality Assurance Program Oversite Laboratory
Expi	expression improvement
FDA	Food and Drug Administration
GMT	geometric mean titer
HEK	human embryonic kidney
HIC	hydrophobic interaction chromatography
IMAC	immobilized metal affinity chromatography
mRNA	messenger ribonucleic acid
MWCO	molecular weight cut-off
NIAID	National Institute of Allergy and Infectious Disease
NIH	National Institutes of Health
QC	quality control
qPCR	quantitative polymerase chain reaction
qSEC	Quantitative Size Exclusion Chromatography
RBD	receptor-binding domain
S-2P	spike with two prolines at amino acid positions 986 and987
SARS-CoV-2	severe acute respiratory syndrome coronavirus 2`
SDS-PAGE	Sodium Dodecyl Sulfate–Polyacrylamide Gel Electrophoresis
SNACs	SARS-CoV-2 Neutralizing Assay Concordance Survey
SpFN	Spike Ferritin Nanoparticle
US	United States
USP	United States Pharmacopeia
VNAR	single-chain variable domain of new antigen receptor
VOC	variants of concern
