# Supplementary material for: Production of Clinical-Grade SARS-CoV-2 Spike Ferritin Nanoparticle Protein Immunogen by Transient Transfection
Source: Vaccines (Basel). 2025 Oct 9;13(10):1041. doi: 10.3390/vaccines13101041 (PMC12567911; doi:10.3390/vaccines13101041)
Supplement: Supplementary file 1 [file vaccines-13-01041-s001.zip › vaccines-3829700-supplementary.pptx]

## Slide 1
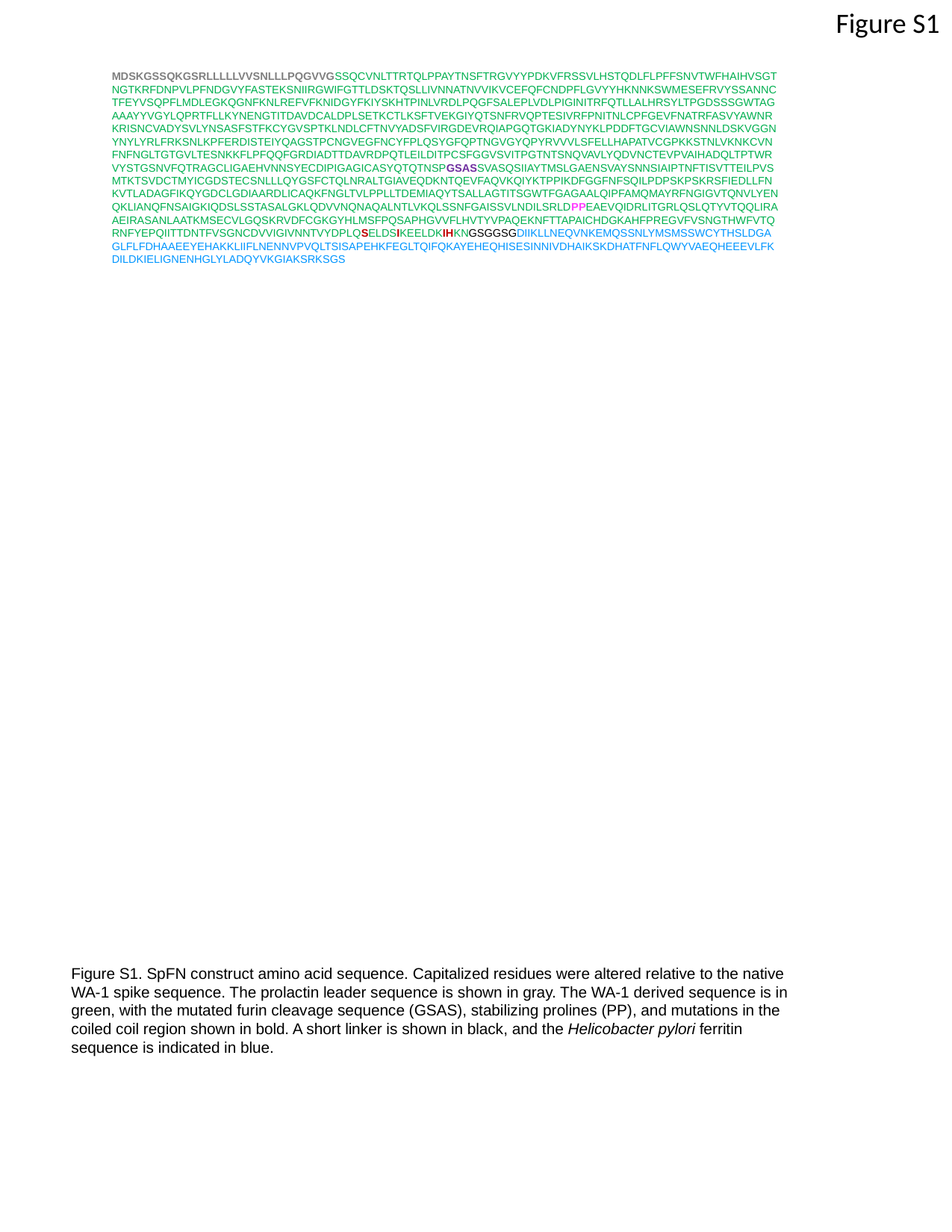

Figure S1
MDSKGSSQKGSRLLLLLVVSNLLLPQGVVGSSQCVNLTTRTQLPPAYTNSFTRGVYYPDKVFRSSVLHSTQDLFLPFFSNVTWFHAIHVSGTNGTKRFDNPVLPFNDGVYFASTEKSNIIRGWIFGTTLDSKTQSLLIVNNATNVVIKVCEFQFCNDPFLGVYYHKNNKSWMESEFRVYSSANNCTFEYVSQPFLMDLEGKQGNFKNLREFVFKNIDGYFKIYSKHTPINLVRDLPQGFSALEPLVDLPIGINITRFQTLLALHRSYLTPGDSSSGWTAGAAAYYVGYLQPRTFLLKYNENGTITDAVDCALDPLSETKCTLKSFTVEKGIYQTSNFRVQPTESIVRFPNITNLCPFGEVFNATRFASVYAWNRKRISNCVADYSVLYNSASFSTFKCYGVSPTKLNDLCFTNVYADSFVIRGDEVRQIAPGQTGKIADYNYKLPDDFTGCVIAWNSNNLDSKVGGNYNYLYRLFRKSNLKPFERDISTEIYQAGSTPCNGVEGFNCYFPLQSYGFQPTNGVGYQPYRVVVLSFELLHAPATVCGPKKSTNLVKNKCVNFNFNGLTGTGVLTESNKKFLPFQQFGRDIADTTDAVRDPQTLEILDITPCSFGGVSVITPGTNTSNQVAVLYQDVNCTEVPVAIHADQLTPTWRVYSTGSNVFQTRAGCLIGAEHVNNSYECDIPIGAGICASYQTQTNSPGSASSVASQSIIAYTMSLGAENSVAYSNNSIAIPTNFTISVTTEILPVSMTKTSVDCTMYICGDSTECSNLLLQYGSFCTQLNRALTGIAVEQDKNTQEVFAQVKQIYKTPPIKDFGGFNFSQILPDPSKPSKRSFIEDLLFNKVTLADAGFIKQYGDCLGDIAARDLICAQKFNGLTVLPPLLTDEMIAQYTSALLAGTITSGWTFGAGAALQIPFAMQMAYRFNGIGVTQNVLYENQKLIANQFNSAIGKIQDSLSSTASALGKLQDVVNQNAQALNTLVKQLSSNFGAISSVLNDILSRLDPPEAEVQIDRLITGRLQSLQTYVTQQLIRAAEIRASANLAATKMSECVLGQSKRVDFCGKGYHLMSFPQSAPHGVVFLHVTYVPAQEKNFTTAPAICHDGKAHFPREGVFVSNGTHWFVTQRNFYEPQIITTDNTFVSGNCDVVIGIVNNTVYDPLQSELDSIKEELDKIHKNGSGGSGDIIKLLNEQVNKEMQSSNLYMSMSSWCYTHSLDGAGLFLFDHAAEEYEHAKKLIIFLNENNVPVQLTSISAPEHKFEGLTQIFQKAYEHEQHISESINNIVDHAIKSKDHATFNFLQWYVAEQHEEEVLFKDILDKIELIGNENHGLYLADQYVKGIAKSRKSGS
Figure S1. SpFN construct amino acid sequence. Capitalized residues were altered relative to the native WA-1 spike sequence. The prolactin leader sequence is shown in gray. The WA-1 derived sequence is in green, with the mutated furin cleavage sequence (GSAS), stabilizing prolines (PP), and mutations in the coiled coil region shown in bold. A short linker is shown in black, and the Helicobacter pylori ferritin sequence is indicated in blue.

## Slide 2
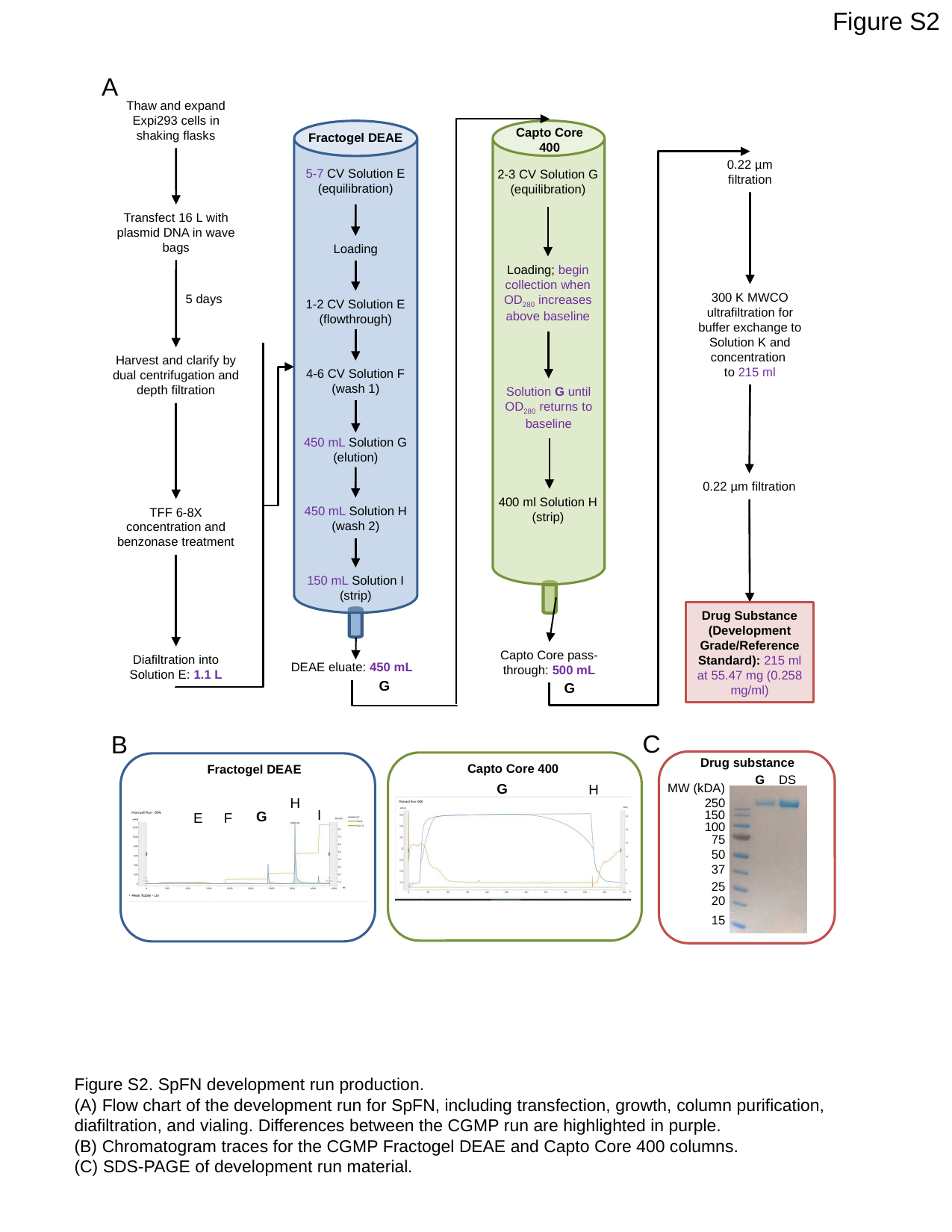

Figure S2
A
Thaw and expand Expi293 cells in shaking flasks
Capto Core 400
Fractogel DEAE
0.22 µm filtration
5-7 CV Solution E (equilibration)
2-3 CV Solution G (equilibration)
Transfect 16 L with plasmid DNA in wave bags
Loading
Loading; begin collection when OD280 increases above baseline
300 K MWCO ultrafiltration for buffer exchange to Solution K and concentration
to 215 ml
5 days
1-2 CV Solution E (flowthrough)
Harvest and clarify by dual centrifugation and depth filtration
4-6 CV Solution F (wash 1)
Solution G until OD280 returns to baseline
450 mL Solution G (elution)
0.22 µm filtration
400 ml Solution H (strip)
450 mL Solution H (wash 2)
TFF 6-8X concentration and benzonase treatment
150 mL Solution I (strip)
Drug Substance (Development Grade/Reference Standard): 215 ml at 55.47 mg (0.258 mg/ml)
Capto Core pass-through: 500 mL
Diafiltration into Solution E: 1.1 L
DEAE eluate: 450 mL
G
G
C
B
Drug substance
G DS
 MW (kDA)
 250
150
100
75
50
37
25
20
15
z
Capto Core 400
Fractogel DEAE
G
H
H
I
G
F
E
Figure S2. SpFN development run production.
(A) Flow chart of the development run for SpFN, including transfection, growth, column purification, diafiltration, and vialing. Differences between the CGMP run are highlighted in purple.
(B) Chromatogram traces for the CGMP Fractogel DEAE and Capto Core 400 columns.
(C) SDS-PAGE of development run material.

## Slide 3
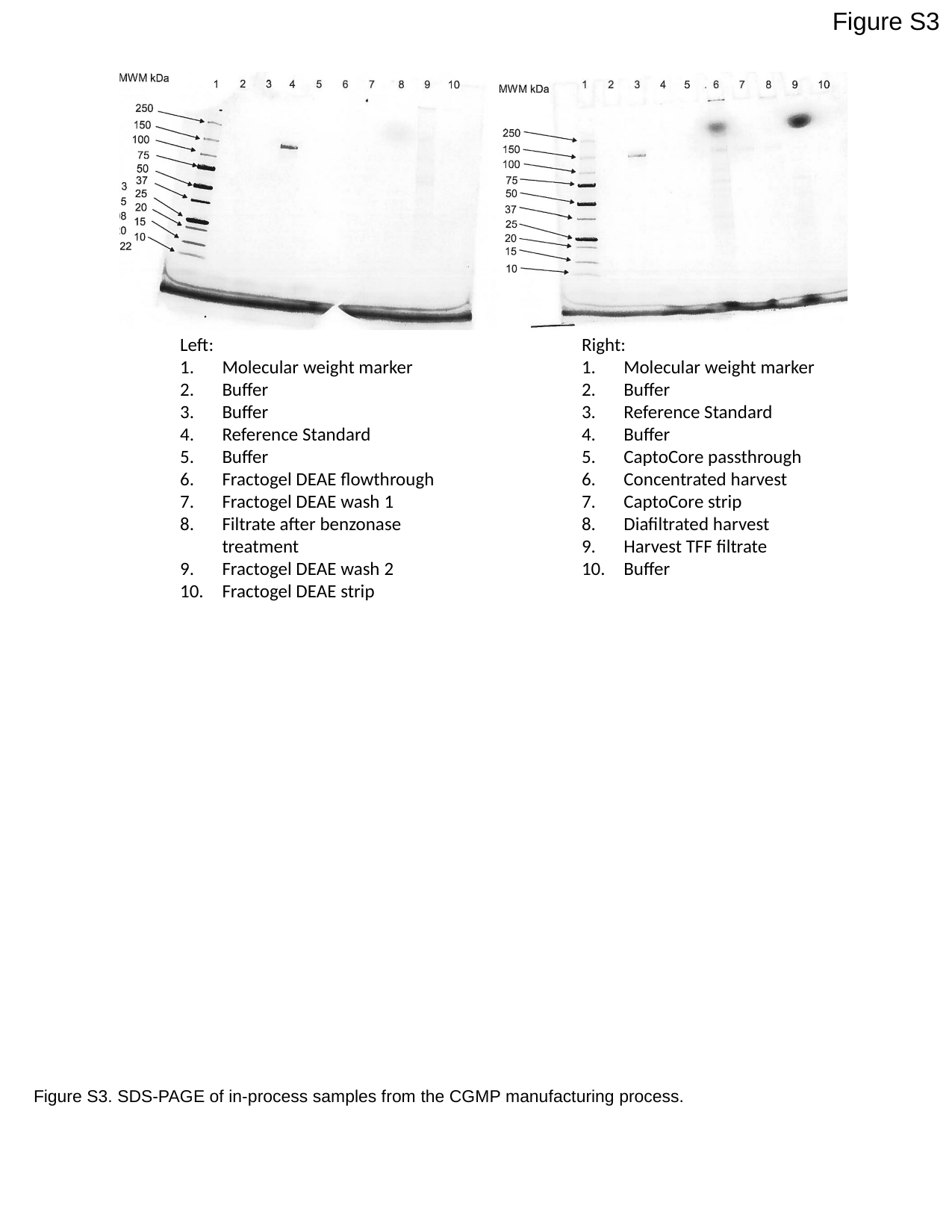

Figure S3
Left:
Molecular weight marker
Buffer
Buffer
Reference Standard
Buffer
Fractogel DEAE flowthrough
Fractogel DEAE wash 1
Filtrate after benzonase treatment
Fractogel DEAE wash 2
Fractogel DEAE strip
Right:
Molecular weight marker
Buffer
Reference Standard
Buffer
CaptoCore passthrough
Concentrated harvest
CaptoCore strip
Diafiltrated harvest
Harvest TFF filtrate
Buffer
Figure S3. SDS-PAGE of in-process samples from the CGMP manufacturing process.

## Slide 4
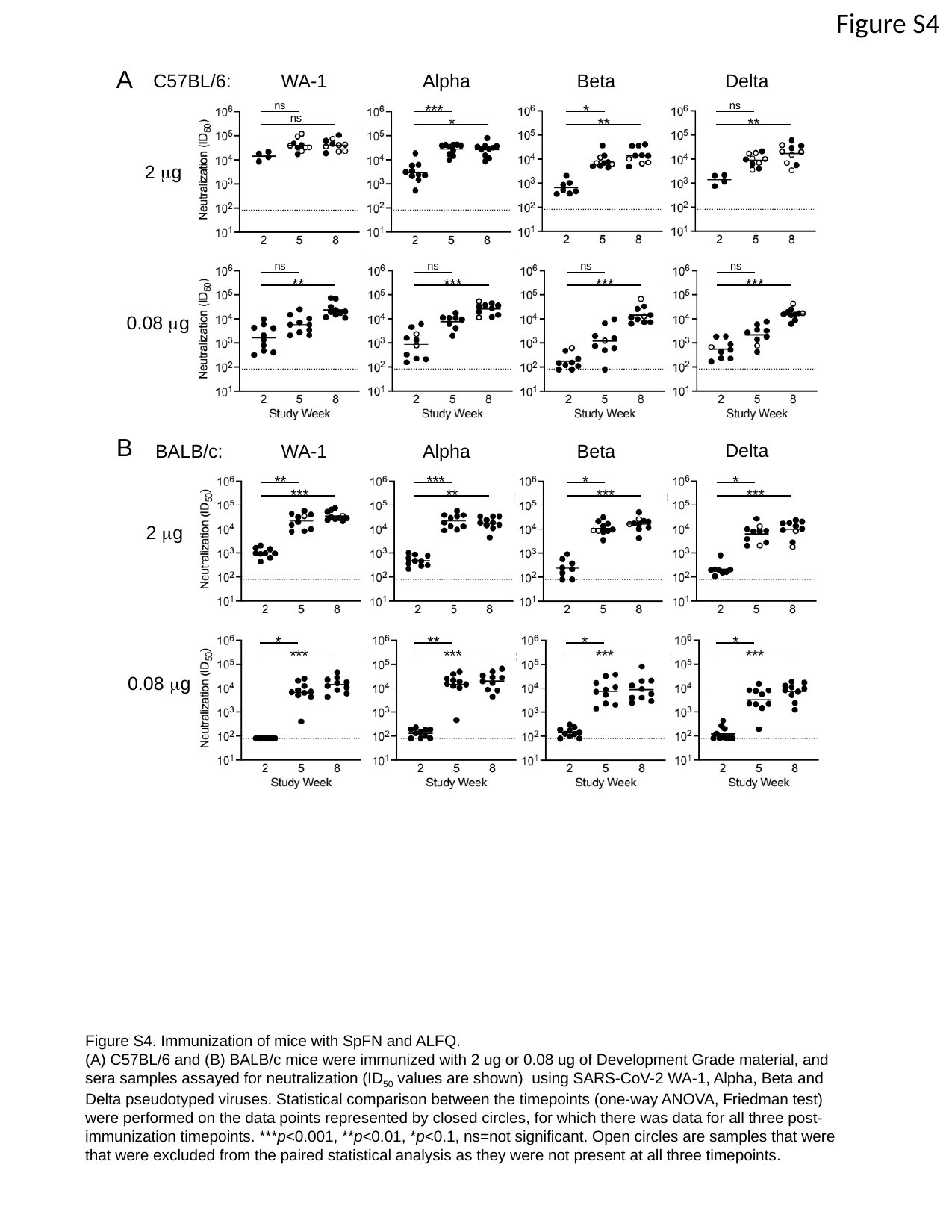

Figure S4
A
Delta
C57BL/6:
Alpha
Beta
WA-1
ns
ns
ns
**
***
*
*
**
2 mg
ns
**
ns
***
ns
***
ns
***
0.08 mg
B
Delta
BALB/c:
Alpha
Beta
WA-1
**
***
***
**
*
***
*
***
2 mg
*
***
**
***
*
***
*
***
0.08 mg
Figure S4. Immunization of mice with SpFN and ALFQ.
(A) C57BL/6 and (B) BALB/c mice were immunized with 2 ug or 0.08 ug of Development Grade material, and sera samples assayed for neutralization (ID50 values are shown) using SARS-CoV-2 WA-1, Alpha, Beta and Delta pseudotyped viruses. Statistical comparison between the timepoints (one-way ANOVA, Friedman test) were performed on the data points represented by closed circles, for which there was data for all three post-immunization timepoints. ***p<0.001, **p<0.01, *p<0.1, ns=not significant. Open circles are samples that were that were excluded from the paired statistical analysis as they were not present at all three timepoints.
